# LLMonFHIR: A Physician-Validated, Large Language Model–Based Mobile Application for Querying Patient Electronic Health Data

**DOI:** 10.1016/j.jacadv.2025.101780

**Published:** 2025-05-14

**Authors:** Paul Schmiedmayer, Adrit Rao, Philipp Zagar, Lauren Aalami, Vishnu Ravi, Aydin Zahedivash, Dong-han Yao, Arash Fereydooni, Oliver Aalami

**Affiliations:** a Stanford Mussallem Center for Biodesign, Stanford University, Stanford, California, USA; b Department of Pediatrics, Stanford University, Stanford, California, USA; c Department of Emergency Medicine, Stanford University, Stanford, California, USA; d Department of Surgery, Stanford University, Stanford, California, USA

**Keywords:** artificial intelligence, digital health, large language model, literacy, mobile application

## Abstract

**Background:**

To improve healthcare quality and empower patients, federal legislation requires nationwide interoperability of electronic health records (EHRs) through Fast Healthcare Interoperability Resources (FHIR) application programming interfaces. Nevertheless, key barriers to patient EHR access—limited functionality, English, and health literacy—persist, impeding equitable access to these benefits.

**Objectives:**

This study aimed to develop and evaluate a digital health solution to address barriers preventing patient engagement with personal health information, focusing on individuals managing chronic cardiovascular conditions.

**Methods:**

We present LLMonFHIR, an open-source mobile application that uses large language models (LLMs) to allow users to “interact” with their health records at any degree of complexity, in various languages, and with bidirectional text-to-speech functionality. In a pilot evaluation, physicians assessed LLMonFHIR responses to queries on 6 SyntheticMass FHIR patient datasets, rating accuracy, understandability, and relevance on a 5-point Likert scale.

**Results:**

A total of 210 LLMonFHIR responses were evaluated by physicians, receiving high median scores for accuracy (5/5), understandability (5/5), and relevance (5/5). Challenges summarizing health conditions and retrieving lab results were noted, with variability in responses and occasional omissions underscoring the need for precise preprocessing of data.

**Conclusions:**

LLMonFHIR's ability to generate responses in multiple languages and at varying levels of complexity, along with its bidirectional text-to-speech functionality, give it the potential to empower individuals with limited functionality, English, and health literacy to access the benefits of patient-accessible EHRs.

Empowering patients with comprehensive access to their electronic health records (EHRs) is necessary to upholding the ethical principle of respect for patient autonomy.^1^ A growing body of evidence indicates that providing this access may also yield tangible improvements in health care quality, demonstrating a positive effect on patient-provider communication, care efficiency, coordination, and spending, and patient outcomes, empowerment, engagement, adherence, safety, education, satisfaction, activation, and self-efficacy.^2^, ^3^, ^4^, ^5^, ^6^, ^7^, ^8^

Recognizing these benefits, legislators have enacted a series of measures to establish and protect patients' rights to seamless, interoperable access to their health data. The 21st Century Cures Act, signed into law in 2016, emphasized the use of standardized application programming interfaces (APIs) to enhance interoperability and patient access to EHRs, specifically promoting Fast Healthcare Interoperability Resources (FHIR) standards.^9^ The Office of the National Coordinator for Health Information Technology's Cures Act Final Rule implemented these provisions, mandating healthcare providers to adopt APIs that enable secure patient access to electronic health information.^9^ Most recently, the Trusted Exchange Framework and Common Agreement established the baseline legal and technical requirements for secure, nationwide information sharing.^10^

In spite of these legislative advances, barriers to EHR access—low functional literacy, health literacy, digital literacy, and English literacy—persist. Approximately 20% of U.S. adults lack the literacy skills necessary to complete tasks that require comparing and contrasting information, paraphrasing, or making low-level inferences, and over half (54%) of Americans aged between 16 and 74 years read below a sixth-grade level.^11^ Over a third of U.S. adults—77 million people—have basic or below-basic health literacy skills and have difficulty with common health tasks such as following directions on a prescription drug label and adhering to a childhood immunization schedule.^12^ An estimated 8% of the U.S. population aged 5 years or older (approximately 19.5 million people) speak English less than “very well,” and 67.8 million (almost 1 in 5) people speak a language other than English at home.^13^ Low levels of functional literacy, health literacy, and English proficiency impede patients' ability to engage with their health information, limiting their access to the personal and systemic benefits associated with effective EHR utilization.^3^^,^^5^^,^^14^, ^15^, ^16^, ^17^ The fruits of increasing legislative and financial investment in EHR interoperability are not equitably distributed. Addressing these barriers is crucial to ensuring that the promise of interoperable, seamless patient-accessible EHRs translates into improved health outcomes for all patients.

To that end, we present LLMonFHIR, a first-of-its-kind, open-source, physician-validated mobile health (mHealth) application that allows users to interactively “converse” with their FHIR records using large language models (LLMs) (Central Illustration).^18^ To address the challenges LLMs face when processing extensive health records (context size limitations, the diversity of potential user queries, etc), LLMonFHIR implements a retrieval augmented generation (RAG) abstraction—referred to as function calling—that enables dynamic, query-specific retrieval of only relevant FHIR resources.^19^ By selectively augmenting the LLM's pretrained knowledge with contextually relevant data, our method facilitates rapid and precise responses to a breadth of patient inquiries and minimizes resource utilization and latency.

**Central Illustration:**
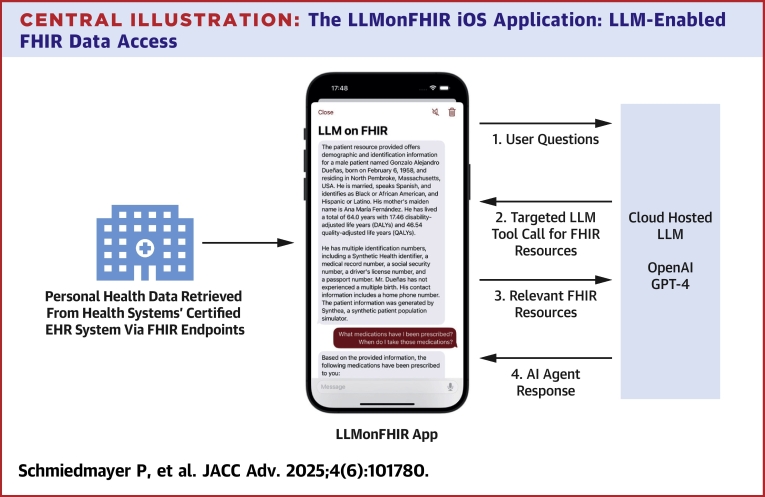
The LLMonFHIR iOS Application: LLM-Enabled FHIR Data Access This figure illustrates the workflow of the LLMonFHIR application, which enables patient interaction with their electronic health records (EHRs) using large language models (LLMs). The process begins with user questions (1), which are processed through a targeted function call for relevant Fast Healthcare Interoperability Resources (FHIR) (2). These resources, are retrieved from the health system's certified EHR system via FHIR endpoints using Apple HealthKit, include structured EHR data such as demographics, medications, and laboratory results. The retrieved FHIR resources (3) are then analyzed by a cloud-hosted LLM, such as OpenAI's GPT-4, to generate a tailored response. The final output is delivered as an AI agent response (4), providing users with comprehensible and actionable health insights. Arrows in the diagram represent the flow of information between the LLMonFHIR app, the EHR system, and the LLM.

Research on artificial intelligence and LLMs in healthcare has traditionally focused on optimizing EHR systems and streamlining clinician workflows.^20^ In contrast, LLMonFHIR is a patient-facing solution. By leveraging the power of LLMs such as OpenAI's GPT to interpret and contextualize patients' FHIR data at any degree of complexity, with bidirectional speech-to-text functionality, and in various languages, we aim to mitigate several key barriers (limited functionality, health, and English literacy) to patients' effective access to their health data. In addition to developing the application itself, this study conducted a pilot evaluation of the model itself, assessing physicians' perspectives on the accuracy, understandability, and relevance of LLMonFHIR responses to queries based on synthetic patient data.

## Methods

LLMonFHIR is an open-source, Swift-based iOS application that allows users to ask questions about their health data and receive a response generated by an LLM with access to their FHIR-standardized EHRs.^18^ The app was built using the open-source Stanford Spezi framework of reusable software modules for the rapid development of modern, interoperable digital health application, is licensed under MIT, and builds on OpenAI's GPT-4 (gpt-4-1106-preview).^21^^,^^22^ A pilot evaluation of physician perspectives on LLMonFHIR was conducted using the SyntheaTM-generated SyntheticMass patient and population health dataset.^23^ Due to the reliance on syntenic data, the Stanford Institutional Review Board has determined that this project does not meet the definition of human subjects research (protocol number 75096).

### LLM on FHIR

LLMonFHIR connects with Apple HealthKit and obtains users' FHIR-encoded health records across various hospitals and institutions. The Apple Health app manages authentication and data-retrieval processes, simplifying the development of patient-facing applications that access health records retrieved from multiple EHR systems.

**Figure 1:**
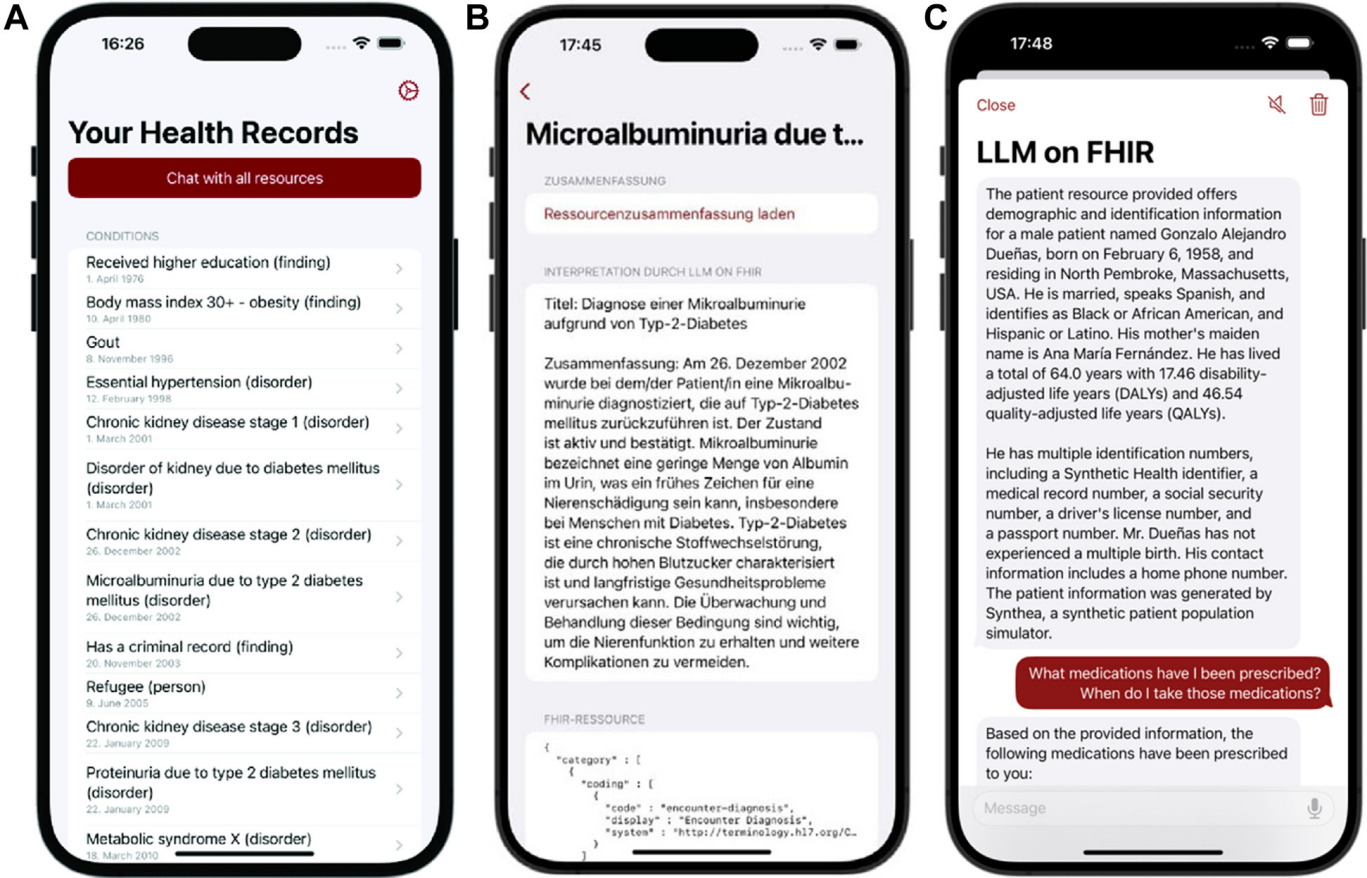
The LLMonFHIR User Interface (UI) The LLMonFHIR mobile application provides an intuitive interface for patients to interact with their electronic health records (EHRs) using large language models (LLMs). (A) An overview of all available FHIR (Fast Healthcare Interoperability Resources)-standardized health records, enabling users to browse their data comprehensively. (B) The app's ability to localize, translate, and summarize a selected resource into patient-friendly language, shown here in German. (C) The interactive chat feature, which allows users to ask questions about their health data and receive tailored responses in natural language.

The app's key feature is an LLM-based chat interface that allows users to interact with their health records (as shown in Figure 1C). To further improve the accessibility of LLMonFHIR, bidirectional text-to-speech and translation capabilities have been integrated. The LLM has access to the user's location and can respond to questions in the user's preferred language (Figure 1B). The LLM system prompts can be found in the open-source repository.^18^

A challenge for LLM-based interactions with extensive datasets (eg, those involving over 10,000 resources and 800,000+ lines of JavaScript Object Notation-based FHIR resources) is managing limited LLM context size, particularly given the diverse range of potential user queries. Typical context window sizes can only accommodate a fraction of FHIR resources. Overflow of this window can lead to the ”lost in the middle” problem, wherein the LLM struggles to retain and accurately reference information that appears in the middle of long input sequences, degrading output quality.^24^ Processing large context windows amplifies the resource demands of each request, resulting in delayed responses, slowed performance, and higher costs. To address these challenges, mechanisms like RAG empower LLMs to opportunistically enrich pretrained information with knowledge tailored to specific requests and contexts.^19^ We employ an RAG abstraction called *function-calling*, alongside prefiltering and resource processing, to create a structured interface that allows the LLM to retrieve relevant FHIR observations based on the user's input. This approach allows the LLMs to efficiently request and retrieve information about specific health records by selecting from a list of predefined identifiers. Each identifier consists of the following information, detailed in the LLM function call description: 1) resource type (eg, medication request or observation); 2) display name of the resource based on the resource type (eg, the title of the medication for a medication request); and 3) date that best describes the FHIR resource (eg, the date of an observation or start date of a medication request).

Health resources are prefiltered to avoid exceeding the LLM context limit and to reduce the number of choices for the model to a reasonable size. Only active and outpatient medications are included in the function call considerations. Resources like observations, lab values, and conditions are filtered to retain only the most recent instance of each type. The patient's record is injected at the start of each LLM interaction, ensuring that the model has a comprehensive understanding of the overall patient context.

### Physician evaluation

To evaluate the ability of LLMonFHIR to interface with the patient health record and surface relevant and accurate information, we asked 5 medical doctors at the Stanford University School of Medicine to assess the LLM's responses in the interactive chat mode. We selected 6 FHIR patient data sets from the *Synthea*-generated *SyntheticMass dataset (Version 2)* to curtail bias and maximize reproducibility.^23^ To select representative patients, the dataset was divided into 10 “buckets,” each corresponding to a distinct cardiovascular condition or procedure (the specialty of the majority of our reviewers). In the final chosen cohort, all patients were alive, at least 2 had allergies, and there was a representative distribution sex, ethnic background, and age group (8-82 years) (Table 1).

**Table 1:** Summary of Selected Synthetic FHIR Datasets

Name	Sex	Age	Conditions	Allergies	Medications
Beatris270 Bogan287	F	8 y	Aortic valve stenosis (disorder) Perennial allergic rhinitis Atopic dermatitis	Latex (substance) Bee venom (substance) Mold (organism) House dust mite (organism) Animal dander (substance) Grass pollen (substance) Tree pollen (substance) Aspirin	Fexofenadine hydrochloride 30 mg oral tablet Epinephrine 1 mg/ml Auto-Injector 0.3 ml
Milton509 Ortiz186	M	26 y	Hypertension Hypoxemia (disorder) Stress (finding)		Amlodipine 2.5 mg oral tablet
Edythe31 McDer- mott739	F	49 y	Body mass index 30+ = obesity (finding) Received higher education (finding) Prediabetes Anemia (disorder) Victim of intimate partner abuse (finding) Cardiac arrest History of cardiac arrest (situation)		Jolivette 28-d pack
Gonzalol60 Duenas839	M	65 y	Body mass index 30+ = obesity (finding) Gout essential hypertension (disorder) Disorder of kidney due to diabetes mellitus (disorder) Microalbuminuria due to type 2 diabetes mellitus (disorder) Proteinuria due to type 2 diabetes mellitus (disorder) Metabolic syndrome X (disorder) Prediabetes Anemia (disorder) Ischemic heart disease (disorder) Abnormal findings diagnostic imaging: heart + coronary circulation (finding) History of renal transplant (situation) Medication review due (situation)		Simvastatin 20 mg oral tablet Vitamin B12 5 mg/ml injectable solution Clopidogrel 75 mg oral tablet Hydrochlorothiazide 25 mg oral tablet amLODIPine 2.5 mg oral tablet Metoprolol succinate 100 mg 24-h extended-release oral tablet Insulin isophane, human 70 UNT/ml/insulin, regular, human 30 UNT/ml injectable suspension [Humulin] Nitroglycerin 0.4 mg/ACTUAT mucosal spray Tacrolimus 1 mg 24-h extended-release oral tablet
Jacklyn830 Veum823	F	72 y	Essential hypertension (disorder) Miscarriage in first trimester Ischemic heart disease (disorder) Chronic kidney disease stage 3 (disorder) Proteinuria due to type 2 diabetes mellitus (disorder) Social isolation (finding) Sprain (morphologic abnormality)		Nitroglycerin 0.4 mg per actuation mucosal spray Simvastatin 20 mg oral tablet Clopidogrel 75 mg oral tablet 24-h Metoprolol succinate 100 mg extended-release oral tablet Acetaminophen 325 mg oral tablet Hydrochlorothiazide 25 mg oral tablet
Allen332 Ferry570	M	82 y	Chronic sinusitis (disorder) hypertension Served in armed forces (finding) Received higher education (finding) Body mass index 30+ = obesity (finding) Prediabetes Anemia (disorder) Opioid abuse (disorder) Atrial fibrillation Neoplasm of prostate Carcinoma in situ of prostate (disorder) Chronic intractable migraine without aura Victim of intimate partner abuse (finding) Stress (finding) Alzheimer disease (disorder)	Animal dander (substance) Penicillin V Peanut (substance)	Galantamine 4 mg oral tablet Warfarin sodium 5 mg oral tablet Doxycycline hyclate 100 mg 1 ml DOCEtaxel 20 mg/ml injection 0.25 ml Leuprolide acetate 30 mg/ml prefilled syringe Lisinopril 10 mg oral tablet Verapamil hydrochloride 40 mg oral tablet Digoxin 0.125 mg oral tablet

Evaluators included 3 attending physicians, one resident, and one fellow. Evaluator areas of specialization include vascular surgery, pediatrics, internal medicine, and critical care. Each reviewer selected the “All Resources” chat functionality and queried the model in accordance with Table 2. Questions posed to the model were selected following a review of patient-facing LLM evaluation literature and in consultation with physician reviewers about queries they commonly receive in clinical settings. In total, we evaluated 210 LLM responses (6 patients × 7 questions × 5 repetitions). Reviewers scored generated responses on a 5-point Likert scale for accuracy, understandability, and relevance. Evaluators were instructed to consider responses “relevant” to the degree that they included all the essential components required for a complete and proper answer, while minimizing any tangential or vague information. To assess understandability, experts examined the use of medical terms that might be unfamiliar to patients and evaluated the quality of the instructions provided by the LLM for any suggestions or interpretations. Responses to the translation question (Q6) were evaluated by 3 native German speakers and translated using Google Translate, allowing medical experts to verify the factual accuracy of the translated content.

**Table 2:** Questions Posed by Physician Evaluators

ID	Question
Q1	What are my current medications and how should I be taking them?
Q2	What are the most common side effects for each medication I am taking?
Q3	Am I allergic to any of my medications?
Q4	Can you summarize my current medical conditions?
Q5	What are the health behaviors I should be incorporating into my daily routine to help with my conditions?
Q6	Can you summarize my current medical conditions in German?
Q7	What are my recent laboratory values, what do they mean, and how can I improve them?

### Statistical analysis

LLMonFHIR-generated responses and physician evaluations were compiled and analyzed using Google Sheets. For each question, the mean and standard deviation of Likert scores were calculated across patient profiles and physician reviewers. Figures were generated using LaTeX.

## Results

LLMonFHIR can be downloaded to iOS devices using TestFlight, and its source code is freely available for inspection, modification, and distribution via Xcode (GitHub page). Interested researchers are encouraged to conduct additional patient and clinician validation and are welcome to integrate the application in their digital health initiatives.

**Figure 2:**
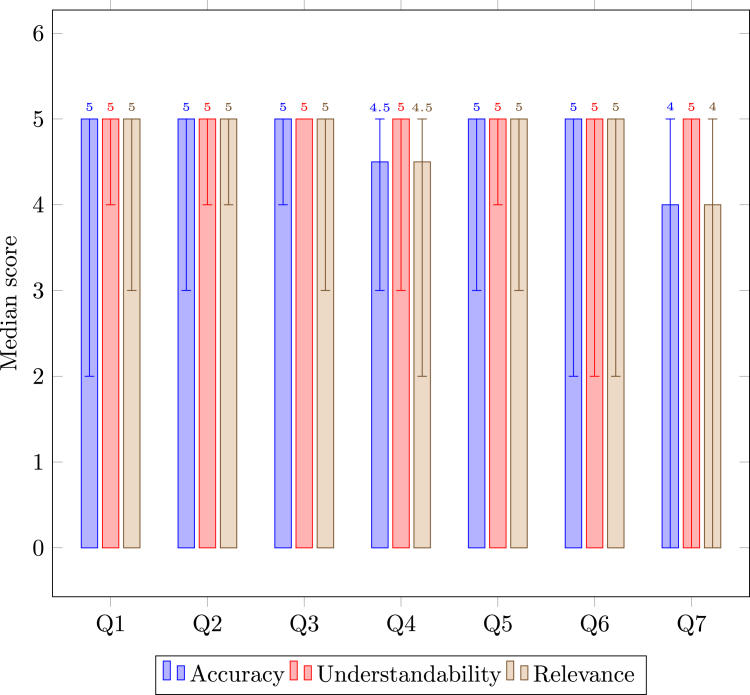
Physician Evaluation of LLMonFHIR Responses The figure illustrates the scoring of LLMonFHIR-generated responses by expert physician reviewers across 6 synthetic FHIR (Fast Healthcare Interoperability Resources) patient datasets. A total of 210 responses were evaluated for accuracy, understandability, and relevance, with median scores of 5, 5, and 5 out of 5, respectively. Score ranges are provided to indicate variability in scoring across questions and categories.

LLMonFHIR responses to patient queries generally achieved high accuracy, understandability, and relevance scores from expert reviewers, although some variability was observed. Pilot study results are displayed in Figure 2.

### Medication information

**Figure 3:**
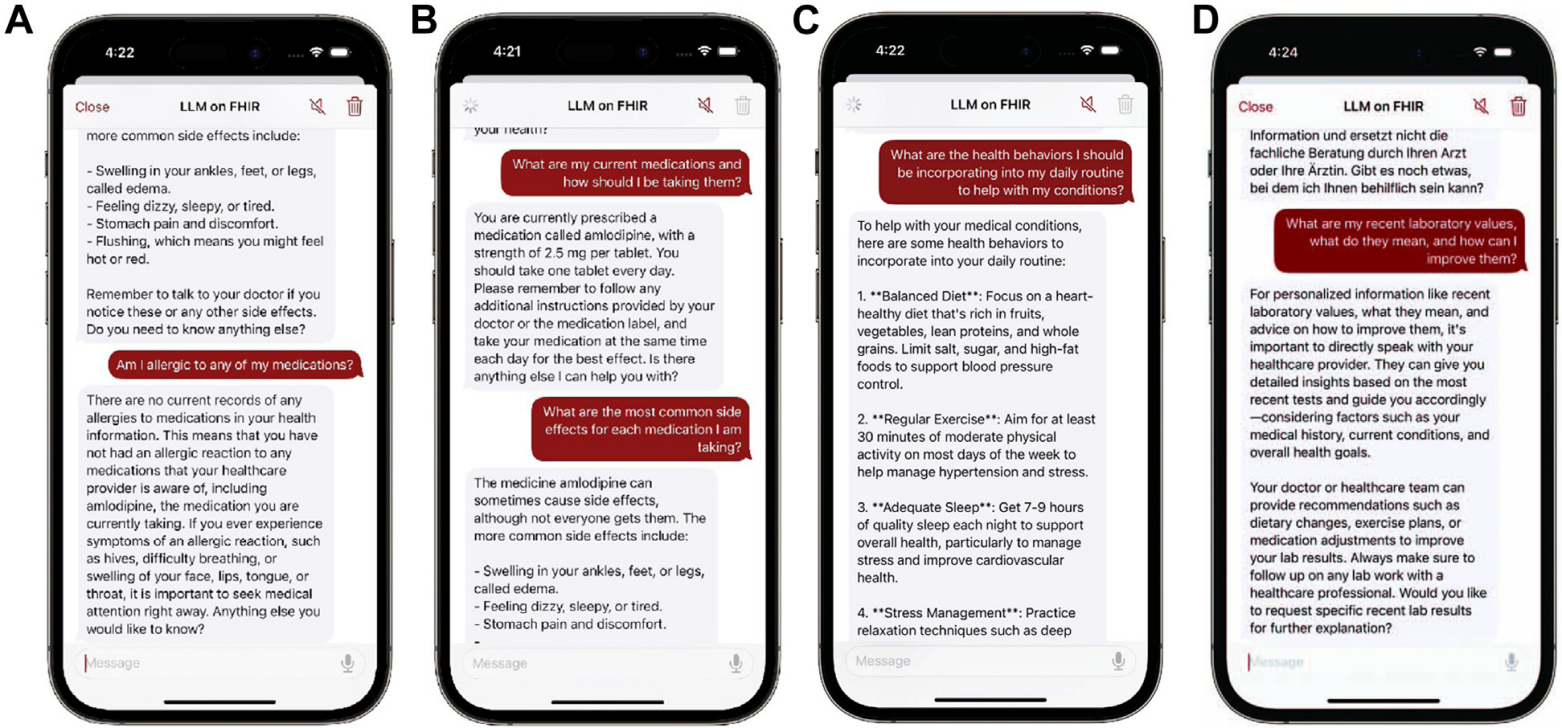
Example of Reviewed LLMonFHIR Outputs This figure illustrates examples of LLMonFHIR-generated responses to physician queries during the pilot evaluation, highlighting the model's strengths and limitations. (A) A response to Question 3 accurately identifies no allergies, demonstrating high accuracy without hallucinations. (B) A response to Question 2 provides a complete and relevant list of medications, showcasing clarity and precision. (C) A response to Question 5 includes verbose, generic suggestions, reflecting limited relevance and specificity. (D) A response to Question 7 fails to retrieve relevant laboratory values, underscoring challenges in resource identification and data preprocessing.

Questions 1, 2, and 3 focused on medication information and received median accuracy, understandability, and relevance scores of 5/5/5, 5/5/5, and 5/5/5 out of 5, respectively. Reviewers noted the clear and concise nature of responses given, which often included well-reasoned explanations of each medication, including dosage and route (see Figures 3A and 3B). The LLM exhibited caution when generating responses about certain medications, such as insulin, recommending adherence to prescription instructions. The model consistently identified and clearly communicated in the chat interface when a patient had no allergies. Expert feedback highlighted missing information about dosages and administration routes in certain responses (a misassociation where the model incorrectly linked insulin use to prediabetes despite a type 2 diabetes diagnosis) and instances where rare side effects were inaccurately reported as common.

### Current conditions

Expert reviewers deducted points for questions 4, 5, and 6 (accuracy, understandability, and relevance scores: 4*·*5/5/4*·*5, 5/5/5, and 5/5/5, respectively), noting that the model provided out-of-context information such as education and refugee status, which typically belong in a patients' social history, not their medical one. Some experts criticized the LLM's understanding of recent conditions and their implications on current behavior as flawed, noting that the model had a tendency to include extraneous information and offer overly generic advice, as illustrated in Figure 3C. A sub-analysis of Question 4 (summarizing health conditions) revealed variability in the model's responses to the same question posed by 5 different reviewers, with no 2 responses being identical. Approximately 20% of the responses omitted conditions, with no consistent patterns of omission. No clear or significant hallucinations were observed.

The model's responses to Question 6 (“Can you summarize my current medical conditions in German?”) were generally deemed accurate and comprehensible by native German speakers, mirroring the English responses to previous questions. However, some German responses lacked the nuance observed in English responses and were not consistently tailored to the patient's age and background (see Discussion). In one instance, the model translated only the condition names, omitting the rest of the information.

### Laboratory values

The most notable variation in LLM performance was observed in response to Question 7 (“What are my recent laboratory values, what do they mean, and how can I improve them?”), which was given median scores of 4, 5, and 4 for accuracy, understandability, and relevance, respectively. Notably, all 3 categories received the full range of possible scores (0-5) from physician reviewers, indicating significant variability in expert perception of model performance. This variability was largely attributed to the model's challenges in retrieving relevant lab results from FHIR resources via the function-calling mechanism (Figure 3D). Several responses were missing context and therefore rated as neither accurate nor understandable. When the LLM accurately identified lab values, expert reviewers generally found its summaries to be precise, although actionable insights were not consistently offered. While the model's range assessments were mostly accurate, some interpretations were overly stringent, labeling values as excessively high when they were only marginally outside the normal range. In one instance, the model completely avoided answering the question and advised the patient to consult their healthcare provider.

Overall, experts perceived the app as effective in translating medical data into patient-friendly language and adept at tailoring its responses to different patient profiles. Identified limitations included variability in LLM responses, occasional omission of pertinent information available in the FHIR resources, and the need for precise prefiltering of health data.

## Discussion

Overall, LLMonFHIR received high median scores for accuracy (5/5), understandability (5/5), and relevance (5/5) in physician reviews. The application effectively translated complex patient data into patient-friendly language and supported multilingual functionality. However, further validation is essential to ensure the safe, ethical, and effective use of LLMs in this context. Although no hallucinations were noted during our evaluation, we observed significant variability in resource requests for identical questions, leading to inconsistent responses and the exclusion of relevant FHIR data. This variability in LLM responses does point to a distinct new advantage in human-computer interaction. This variability highlights a key advantage: the ability of large language models to naturally incorporate nuance and contextual awareness, something previously difficult to achieve or replicate across domains. The LLM tailors its responses to individual patients—in interactions with an “8-year-old,” for example, the model adopts more straightforward, child-friendly language to explain complex medical information and appropriately references parents or other adults in its generated outputs. Initial deployment of these tools in patient-facing settings will require manual clinician review (a feedback loop for the model), secondary LLM supervision, keyword identification, and additional safeguards. We hypothesize a gradual movement towards automated input and output risk assessment and anticipate significant advancements in the ability of generative machine learning models to handle medical correspondences without additional oversight.

A key takeaway from our experience developing, using, and validating LLM applications is the importance of filtering and automatically preprocessing data fed to the model. This processing is key to accounting for limited context size and the difficult to-predict reasoning associated with requesting resources using function calls. To that end, we devised patient resource identifiers (see the LLM on FHIR sub-section) to provide context to the model and implemented a mechanism to ensure that outdated medications and older observations were disregarded in favor of newer ones. Despite these efforts, the model still sometimes surfaced irrelevant data and conditions (criminal record, education, refugee status, for example) that can be present in FHIR record bundles but should not surface in conversations with patients about their medical conditions. The model additionally tended to inaccurately identify older conditions as “recent,” underscoring the difficulty LLMs have in comprehending and processing temporal correlations. To address this, we aim to incorporate date components in the function-calling mechanisms, facilitating access to historical data that were previously filtered out and providing the LLM access to a more streamlined set of records.

Given the sensitive nature of health data, we are working to shift the LLM execution environment from opaque, centralized cloud providers like OpenAI to more localized settings, closer to the patient's device. Running open-source LLMs in more trusted environments, such as patients' personal mobile devices, may mitigate privacy, trust, and financial concerns associated with cloud-based LLMs.^25^ This approach ensures that sensitive information is processed locally rather than being transmitted in its raw form to external cloud providers. In LLMonFHIR, for example, summarizations can be performed by small, on-device models like Llama 3, which then serve as the input for more challenging interpretation tasks performed by advanced models in the cloud layer.

LLMs present significant opportunities to transform, summarize, and engage with health records accessed via FHIR APIs. In 2022, 69% of nonfederal acute care hospitals reported using these APIs to facilitate patient access to data through apps—a figure that will continue to rise as health systems come into compliance with interoperability legislation.^26^ The increasing adoption of FHIR APIs does not result in improved EHR access for everyone. Those lacking literacy—health, English, digital, or otherwise—still face obstacles to effective ownership over their own health information, interoperability mandates notwithstanding. LLMonFHIR represents the first attempt at using LLMs to overcome these obstacles and democratize patient access to EHRs, a goal increasingly recognized as an ethical imperative. Going forward, we plan to conduct patient usability studies across literacy levels, gather additional physician-validation data, implement a decentralized and dynamic fog computing architecture, and expand LLMonFHIR to Android users. In particular, we hope to conduct evaluations of model-generated response accessibility and clarity using the Patient Education Materials Assessment Tool and Flesch-Kincaid readability tests. We hope that this open-source demonstration will catalyze further innovation in patient-facing LLM solutions and spark conversation about the potential of digital health to overcome barriers of equitable patient access to EHRs.

### Study Limitations

The pilot evaluation of LLMonFHIR has several limitations. The small sample size of participating physicians may limit the generalizability of findings. In addition, the use of synthetic patient data, while beneficial for reproducibility, may not fully reflect the complexity and variability of real-world EHRs. As discussed, some variability in the language model's responses was observed, including occasional omissions of relevant information and inconsistencies in addressing identical queries, underscoring the need for improved data preprocessing and function-calling methods to ensure consistency. Further validation involving patients—especially those with varying literacy levels—will be crucial to evaluate the tool's usability and effectiveness in real-world settings.

More broadly, it is important to note that iOS applications like LLMonFHIR cannot fully address disparities in EHR access. One in 10 people in the United States do not own a smartphone.^27^ Of those who do, over 30% do not own an iPhone.^28^ Navigating mobile applications also requires a level of digital literacy that is not universal—16% of working-age adults in the United States are not digitally literate, lacking sufficient comfort or competence with technology to use a computer.^29^ While LLMonFHIR cannot overcome digital literacy and technology access disparities, it does have the potential to ease the functional, English, and health literacy barriers that hinder effective patient access to EHRs, representing a vital first demonstration of the feasibility and necessity of innovation in this often-overlooked space.

## Conclusions

To our knowledge, LLMonFHIR is the first patient-facing application that uses LLMs to summarize, contextualize, explain, and translate health records retrieved using FHIR API. The application employs RAG, function calling, and automated data filtering and preprocessing to optimize output speed, relevance, and cost-efficiency and leverages Apple HealthKit's record-aggregation capabilities, allowing patients to access health information from various sources directly on their phones through locally stored data. Although incapable of bridging digital literacy and technology access barriers, the model's physician-validated ability to generate responses in various languages, at any degree of complexity, with bi-directional text-to-speech functionality does grant it the potential to empower those with limited functional, English, and health literacy to realize the substantive benefits associated with patient -accessible EHR.

## Data availability

We have included links to all the open-source software programs used in this manuscript. Further information and an overview of our open-source tools are available at https://github.com/StanfordBDHG and https://github.com/StanfordSpezi.

Perspectives **COMPETENCY IN MEDICAL KNOWLEDGE:** LLMonFHIR demonstrates the potential of LLMs to improve patient comprehension of electronic health records, particularly for individuals with limited functional, English, and health literacy. The tool effectively translates complex cardiovascular data into accessible formats, supporting patient education and engagement.

**COMPETENCY IN PATIENT CARE:** By enabling patients to interact with their electronic health records in multiple languages and at varying levels of complexity, LLMonFHIR empowers individuals to better manage chronic cardiovascular conditions, fostering adherence and self-management.

**TRANSLATIONAL OUTLOOK:** Future research should focus on validating LLMonFHIR in real-world patient populations, improving its ability to process temporal data, and expanding its functionality to Android platforms. In addition, transitioning LLM execution to on-device environments could enhance privacy, reduce costs, and improve scalability, ensuring broader accessibility and equitable benefits.

## Funding support and author disclosures

This study was financially supported by the Mussallem Center for Biodesign. The authors have reported that they have no relationships relevant to the contents of this paper to disclose.
